# The Editor's Letter-Box

**Published:** 1887-11-26

**Authors:** 


While fully agreeing with "Sister Mina Lee" that time on duty is as a rule too long for sisters and nurses, allow me to quote St. Mary's Hospital, Paddington, as a grand exception to this rule. It would be well if other hospitals adopted the admirable arrangement for "time off duty" so splendidly organised by the former matron, Miss Rachel Williams, and which is still carried on at St. Mary's. "Sisters" have from five p.m. till half-past ten p.m. twice a-week; seven p.m. to half-past ten p.m. once a-week, and alternate Sundays from three p.m. till ten p.m. "Staff nurses" the same time, but commencing half-past four instead of five. Each has a day off once a month. Probationers, two hours "off duty" each day, and alternate Sundays from three p.m.; a day off once a month. The fact of not having to return on duty three evenings a-week was in itself a rest to mind and body, and most fully appreciated by sister and nurse.

One who was on the Staff During Miss Williams's Time.

